# Kinetics of Wnt-Driven β-Catenin Stabilization Revealed by Quantitative and Temporal Imaging

**DOI:** 10.1371/journal.pone.0003498

**Published:** 2008-10-22

**Authors:** Rami N. Hannoush

**Affiliations:** Department of Protein Engineering, Genentech Inc., South San Francisco, California, United States of America; Instituto de Tecnologia Química e Biológica, Portugal

## Abstract

The Wnt/β-catenin signal transduction pathway regulates a broad range of developmental processes. Aberrant activation of the Wnt pathway leads to cancer and degenerative diseases. β-catenin is a key signaling molecule that is frequently used as a direct monitor of Wnt pathway activation. This paper describes a multi-parametric method for quantitative analysis of cellular β-catenin protein levels in a rapid and high-throughput manner. The assay offers temporally resolved detection of Wnt-stimulated accumulation of β-catenin, simultaneously detecting cell number, and it sheds light onto the kinetics of posttranslational stabilization of β-catenin.

## Introduction

The Wnt/β-catenin canonical pathway plays key roles in development and in the progression of several types of human cancer [1], [2], [3]. β-catenin is an important positive regulator of this signaling pathway, and is dephosphorylated and subsequently stabilized upon binding of Wnt ligands to the cell surface. A consequence of this increase in cytoplasmic β-catenin levels is the concomitant translocation of β-catenin to the nucleus where it interacts with transcriptional coactivators to initiate transcription of Wnt target genes. Since β-catenin is critical for relaying the Wnt signal, researchers frequently quantify total cellular levels of β-catenin to measure Wnt response or to detect perturbations of the pathway. The standard detection method relies on gel electrophoresis coupled with western blotting to quantify total endogenous protein. The limitations of this method are that it is low-throughput, requires large number of cells and contains inherent variability due to gel lane loading, thus hindering the rapid parallel analysis of multiple samples. Other methods for detecting β-catenin stabilization rely on the expression of β-catenin fused to genetically-encoded reporters such as green fluorescent protein (GFP) or luciferase [4], [5], but these may result in artifacts due to β-catenin over expression. Cellular toxicity due to transfection is another frequently encountered problem.

## Results and Discussion

**Figure 1:**
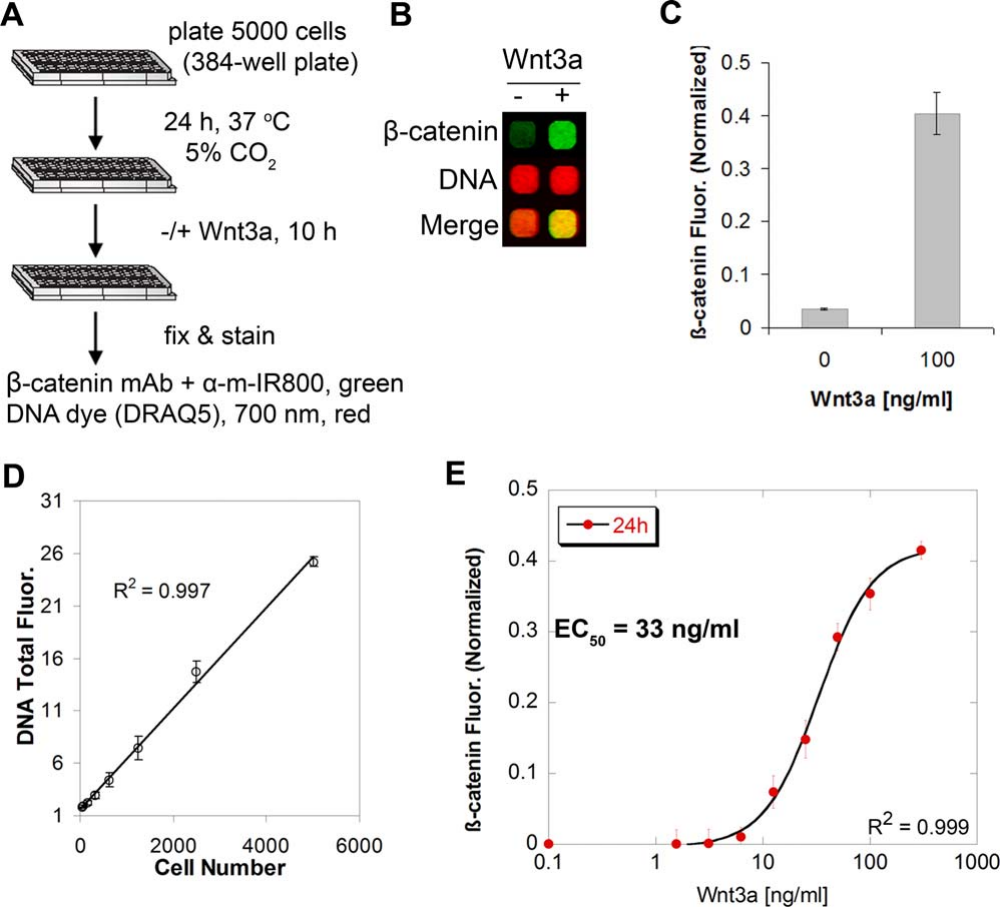
Description and validation of a quantitative method for temporal detection of cellular β-catenin. (A) Schematic of the assay. m denotes mouse species. (B) A representative output infrared image of L-cells treated with or without Wnt3a (100 ng/ml) for 10 h. The image shows whole-cell fluorescence from actual wells of a 384-well plate, highlighting increase in β-catenin signal (green, 700 nm) without change in cellular DNA content (red, 800 nm, detected by DRAQ5). (C) Quantification of the β-catenin signal from (B). The signal is background subtracted and normalized to DNA fluorescence. Values represent the mean±SEM from quadruplicate experiments. (D) Linearity of measured DNA fluorescence (DRAQ5 signal) versus actual number of L-cells plated per well. (E) Dose titration of Wnt3a at 24 h illustrating half-maximal activation of cellular β-catenin in L-cells occurs at 33 ng/ml ligand.

I developed a rapid, quantitative and high-throughput method for measuring Wnt-induced changes in endogenous β-catenin levels. The assay employs mouse L-cells because they are an established model system for studying Wnt signaling [6]. β-catenin in L-cells is predominantly distributed in the cytoplasm and is not present at cell junctions, allowing direct measurement of changes in Wnt-responsive cytoplasmic β-catenin pools. After seeding in a 384-well plate at a density of 5000 cells/well for 24 h, cells were stimulated with purified Wnt3a ligand for 10 h at 37°C. The cells were then fixed and processed for staining with fluorophores in the infrared range to simultaneously detect β-catenin and nuclei (**Figure 1A**). Anti-β-catenin antibody concentration was optimized for best signal to background ratio (see methods), detected by secondary IR800 antibody (800 nm). DNA staining with DRAQ5 dye [7] (700 nm) was used as a marker to normalize β-catenin signal for cell number, particularly important for monitoring a pathway known to affect proliferation [8]. Whole-cell fluorescent images of entire wells were acquired at low resolution at 700 nm (red) and 800 nm (green) (**Figure 1B**). L-cells stimulated with Wnt3a ligand exhibit 11-fold elevated levels of total β-catenin compared to non-stimulated cells without changes in the total DNA content as shown by an increase in the green signal, making the merged signal more yellow than red (**Figure 1B, C**). The assay is highly reproducible with a standard deviation within 10% of the mean of replicates (n = 4). Moreover, there is an excellent linear correlation (R^2^ = 0.997) between the measured DNA fluorescence and the actual number of cells plated in each well (**Figure 1D**), thus allowing for sensitive monitoring of fluctuations in cell number under various treatment conditions. Because the assay is conducted in a 384-well plate format, it minimizes reagent usage and is readily scalable. For example, an entire dose response curve of Wnt3a in quadruplicate can be obtained in 6 h using one 384-well plate (**Figure 1E**). All of this is in sharp contrast to the standard method of preparing individual lysates for analyzing β-catenin by western blotting.

**Figure 2:**
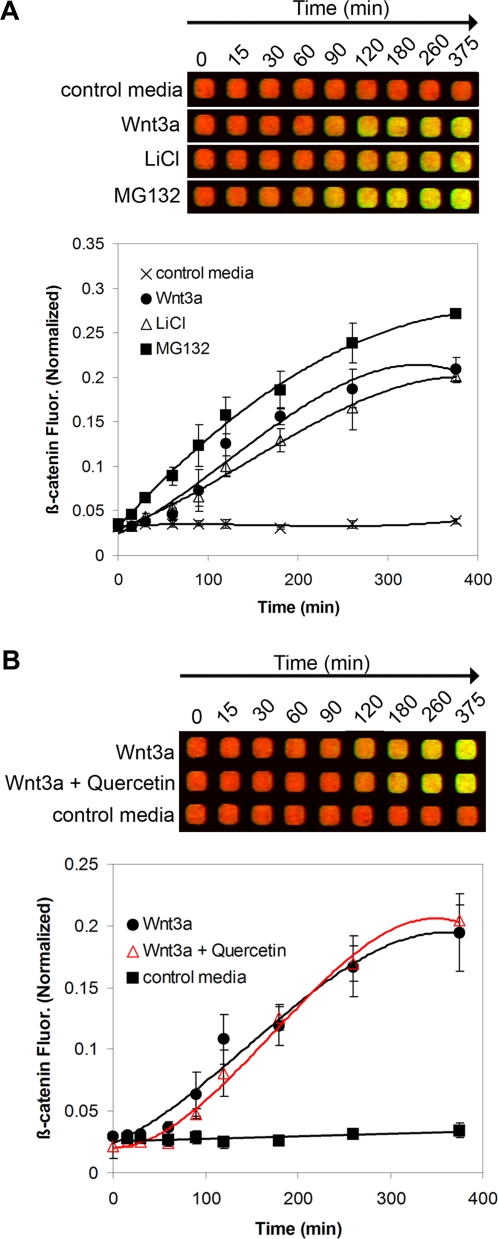
Monitoring cellular β-catenin accumulation in response to various stimuli. (A) L-cells were treated with either Wnt3a (50 ng/ml), LiCl (50 mM) or MG132 (25 µM) for the indicated periods of time and processed for imaging as described in methods. (B) Monitoring the effects of a pharmacologic inhibitor of Wnt signaling on β-catenin accumulation. L-cells were treated with Wnt3a (50 ng/ml) in the absence or presence of 100 µM quercetin for the indicated times. Each point represents mean±SEM (n = 3–4).

To validate the sensitivity of the assay, intracellular β-catenin levels were measured in response to various treatments known to modulate the Wnt/β-catenin pathway. GSK3β is a serine-threonine kinase involved in β–catenin phosphorylation, leading to its degradation [9], [10]. L-cells treated with the GSK3β inhibitor LiCl (50 mM) [11] or the proteasome/calpain inhibitor MG132 (25 µM) [12] showed a marked increase in β-catenin fluorescence within 30–60 min (**Figure 2A**). L-cells treated with Wnt3a (100 ng/ml) showed a similar profile of time-dependent β-catenin accumulation (**Figure 2A**). As expected, treatment with Wnt3a in the presence of quercetin (100 µM), a natural product that inhibits Wnt/β-catenin signaling without altering cytosolic β-catenin levels [13], had no effect on time-dependent β-catenin response (**Figure 2B**). Similarly, treatment with Wnt3a in the presence of the Wnt inhibitor ICG-001 (10 µM) [14] that acts downstream of β-catenin had no significant effects on β-catenin stabilization as expected (**Figure S1**). These examples illustrate the power of our assay to temporally resolve intracellular levels of β-catenin under various conditions, and to readily detect the fast response of β-catenin to GSK3β or proteasome inhibitors at relatively short treatment times. In summary, this new method is versatile and can be readily adapted to a high-throughput format. It can also be utilized for assessing the cellular effects of inhibitors of GSK3β on β-catenin accumulation, or for screening compounds for Wnt pathway inhibition.

**Figure 3:**
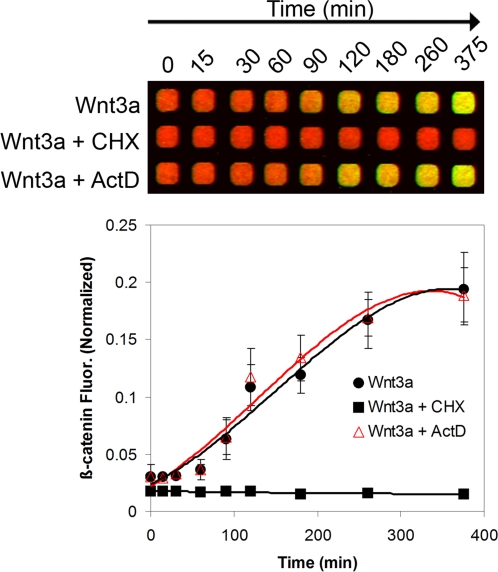
Dependence of Wnt3a-driven accumulation of cellular β-catenin on new protein but not RNA synthesis. L-cells were treated with Wnt3a (50 ng/ml) in the absence or presence of either cycloheximide (CHX, 100 µg/ml) or actinomycin D (ActD, 1 µM) and incubated for the indicated times (n = 4).

To determine if the increase in β-catenin fluorescence upon Wnt3a addition reflects posttranslational stabilization of β-catenin or new protein synthesis, L-cells were treated with either cycloheximide, a protein synthesis inhibitor, or actinomycin D, a DNA transcription inhibitor. Over 6 h, Wnt3a-dependent accumulation of β-catenin shows an almost indistinguishable profile in the absence or presence of actinomycin D, but is severely impacted upon treatment with cycloheximide (**Figure 3**). This shows that protein translation is absolutely required for Wnt-mediated accumulation of β-catenin; it also suggests that in the early phase of Wnt3a activation, stabilized β-catenin originates from existing mRNA pools and is not translationally derived from newly transcribed mRNA.

**Figure 4:**
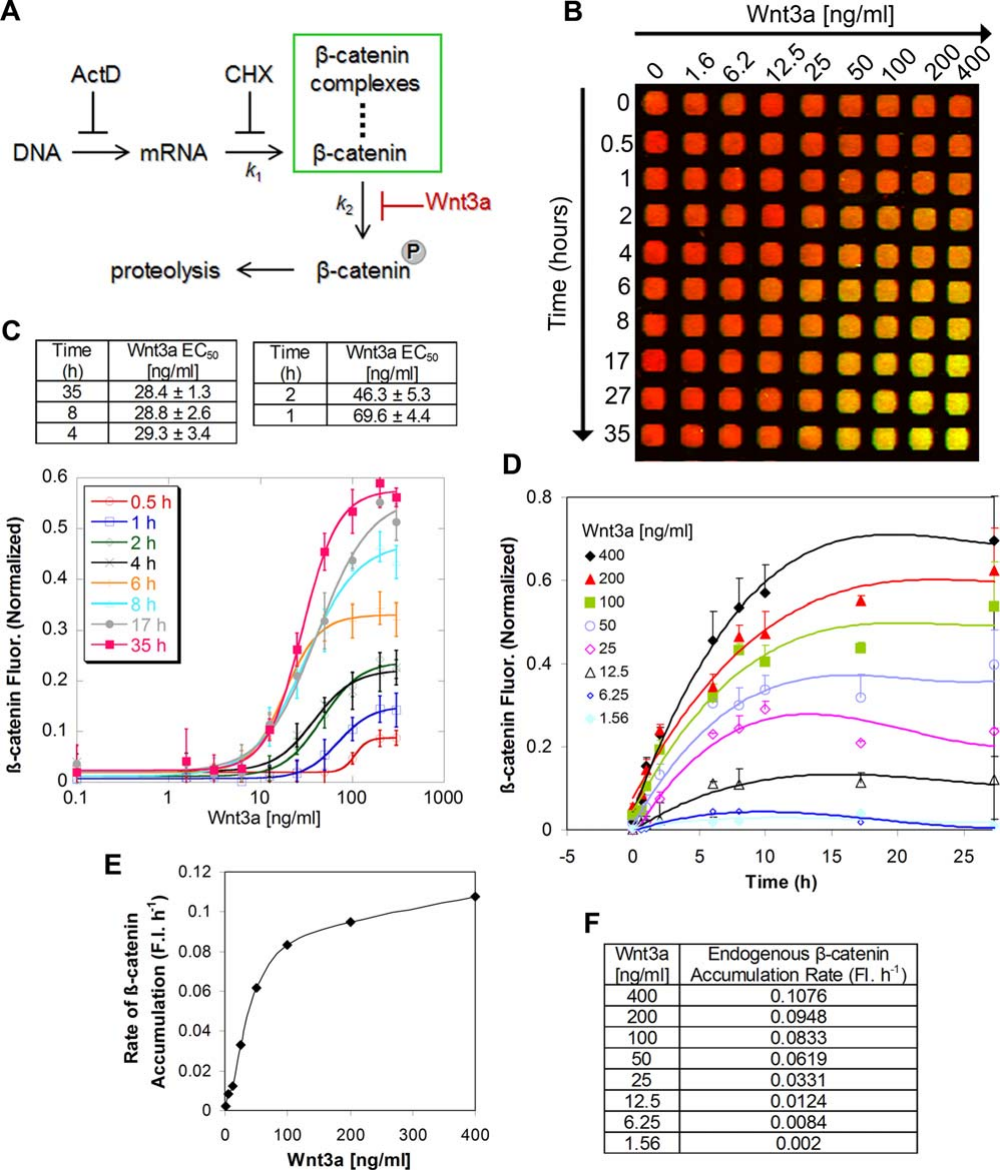
Kinetics of cellular β-catenin accumulation upon stimulation with Wnt3a. (A) Kinetic model of cellular β-catenin accumulation. The model assumes negligible β-catenin turnover in the presence of Wnt3a activation. *K*_1_ and *K*_2_ denote the rate constants of β-catenin translation and phosphorylation, respectively. (B) Time- and dose-dependent accumulation of cellular β-catenin. L-cells were incubated with varying concentrations of Wnt3a for the indicated times. (C) Dose titration of Wnt3a at various time points. The curves are fitted to a non-linear variable slope sigmoidal equation (*R* ^2^ values 0.990–0.999). (D) Kinetics of β-catenin accumulation at different Wnt3a concentrations. The curves are fitted to a non-linear polynomial equation (*R* ^2^ values 0.970–0.999). (E, F) Initial rates of cellular β-catenin accumulation as a function of Wnt3a concentration. Rates were calculated from slopes of the fitted lines during the initial 2 h of treatment. FI. h^−1^ denotes fluorescence intensity per hour. All plots are representative of two independent experiments, each carried out in quadruplicate.

Little is known about the experimental rate of accumulation of endogenous β-catenin upon Wnt activation in mammalian cells. To develop a better quantitative understanding of the Wnt/β-catenin pathway, I determined the initial rate of accumulation of cellular β–catenin upon Wnt pathway activation and studied in detail its dependence on Wnt3a concentration in L-cells. A simplified kinetic model [15] of the Wnt/β-catenin pathway depicts that under basal conditions β-catenin is degraded due to a predominantly high rate of phosphorylation (**Figure 4A**). The presence of Wnt3a inhibits phosphorylation of β-catenin, thus stabilizing β-catenin and preventing its proteasomal degradation (**Figure 4A**). The model assumes that the intrinsic turnover rate of β-catenin through mechanisms other than phosphorylation and that are independent of Wnt is minimal. First, the effective Wnt3a concentration (EC_50_) for half-maximal accumulation of intracellular β-catenin was calculated and shown to depend on the period of stimulation (**Figure 4B, C**). For example, the EC_50_ is ∼30 ng/ml for 4 h–35 h and ∼50–70 ng/ml for only 1 h–2 h stimulation with Wnt3a (**Figure 4C**). These measurements define the sensitivity of the assay and establish optimal conditions for usage. Furthermore, β-catenin levels are upregulated within 30 mins after Wnt3a stimulation, exhibit a noticeable increase in intensity between 6–8 h, and start to plateau after 10 h (**Figure 4D**). The rate of β–catenin accumulation reflects its rate of cellular translation, which varies according to the concentration of Wnt3a presented to cells. At Wnt3a concentrations lower than ∼50 ng/ml, there is a linear correlation between initial rate and Wnt3a concentration (**Figure 4E**); for example, the rate is 0.002 FI. h^−1^ at 1.6 ng/ml Wnt3a and increases by 4-fold upon quadrupling the concentration of Wnt3a (**Figure 4F**). However, at Wnt3a concentrations higher than ∼50 ng/ml, this linear relationship is lost as intracellular β-catenin starts to reach steady-state pools (see inflection and plateau in **Figure 4E, F**). In summary, the method reveals an unprecedented level of detail about the rate of accumulation of intracellular β-catenin, and is useful for deriving quantitative kinetic parameters about the Wnt/β-catenin pathway that may serve as basis for future kinetic modeling of the pathway [15]. Further investigation into the kinetic rate of β-catenin degradation is beyond the scope of this paper.

In conclusion, probing Wnt signaling at the cellular level in a quantitative manner is essential to understanding the signaling cascades implicated in this pathway. The described multi-parametric method is a rapid way to quantify levels of endogenous β-catenin and identify non-specific cytotoxic conditions that result in artifactual readouts. The assay is high-throughput and economical since it is conducted in a 384-well plate format and hence utilizes very small amounts of the expensive purified Wnt3a material. It also offers reproducibility and great speed compared to gel electrophoresis and western blotting. It can easily be used as a secondary screening assay to quickly assess whether pharmacologic inhibitors of Wnt/β-catenin signaling act upstream or downstream of β-catenin. For example, the Wnt inhibitor quercetin has no effect on Wnt-mediated β-catenin accumulation, indicating that it acts downstream of β-catenin. The limitation of the current method, however, is that it lacks spatial resolution and thus does not provide information on the cytoplasmic or nuclear localization of β-catenin nor its translocation behavior. Nevertheless, this assay is a quick diagnostic readout for (1) screening and validating the mechanism of action of modulators of β-catenin or GSK3β, and (2) quantifying the basal and Wnt-induced expression levels of β-catenin across different cell types [16]. In addition, this method offers temporal dynamic resolution and thus can be used to measure the kinetic rate of β-catenin translation as well as turnover and stabilization.

## Materials and Methods

### Reagents

Mouse fibroblast L-cells were obtained from ATCC, maintained as per the manufacturer's recommendations in growth medium (DMEM/10% fetal bovine serum) in an incubator at 37°C and 5% CO_2_. Cell-Bind™ 384-well plates and monoclonal anti-β-catenin antibody [cat # 610154] were purchased from Becton-Dickinson (San Jose, CA). Anti-mouse IRDye800CW and DRAQ5 (far-red) were obtained from Rockland (Gilbertsville, PA). All chemicals were purchased from Sigma (St. Louis, MO). ICG-001, prepared according to literature precedence [14], was from Janet Gunzner (Genentech Inc.). Mouse Wnt3a, purified from mouse L-cells as described earlier [17], was obtained from James Ernst (Genentech Inc.).

### Cellular β-catenin Assay

On Day 1, L-cells (5000 cells/20 µl/well) were seeded in a clear bottomed, black walled 384-well plate and grown for 24 h at 37°C/5% CO_2_ (The method quantitatively detects β-catenin levels in other cell lines such as SW480, HCT15 (colorectal cancer) and U2OS (osteosarcoma); **Figure S2**. Cell densities are as follows: SW480: 6000 cells/well, U2OS: 5000 cells/well, HCT15: 6000 cells/well). On Day 2, the cells were treated with 20 µl of a 2× stock of Wnt3a dissolved in growth medium at the indicated concentration and then incubated for the desired amount of time at 37°C/5% CO_2_. For treatment with various stimuli or inhibitors, 20 µl of 2× stock of chemicals dissolved in growth medium such that the DMSO concentration did not exceed 0.5% were added to the wells and then incubated for the desired amount of time. The cells were then fixed in 4% PFA by adding 20 µl of 12% PFA directly to the wells for 1 h at room temperature. The wells were washed three times with PBS (50 µl/well), permeabilized with PBS/0.1% Triton X-100 (50 µl/well, three times, 2 mins each), and blocked in LI-COR buffer (50 µl/well) for 2 hours at room temperature (or alternatively overnight at 4°C). The wells were then incubated with mouse anti-β-catenin antibody (1: 200 for optimal signal-to-noise ratio) in LI-COR blocking buffer for 2 hours at room temperature (20 µl/well) and subsequently washed with PBS/0.1% Tween-20 (50 µl/well, three times). Infrared anti-mouse IRDye800CW secondary antibody (1: 200) and DRAQ5 (1: 10,000) in PBS/0.5% Tween-20 were then added (20 µl/well). The plates were incubated for 1 hour at room temperature, and the wells were washed with PBS/0.1% Tween-20 (three times) and incubated in PBS (50 µl/well). The plates were covered with black seals and imaged on an Odyssey infrared scanner using microplate2 settings with sensitivity of 5 in both the 700 and 800 nm wavelength channels. Data were acquired by using Odyssey software, exported and analyzed in Excel (Microsoft, Redmond, WA) or KaleidaGraph (Synergy Software). β-catenin values were background subtracted from wells treated only with secondary antibody, and then normalized to cell numbers by dividing by the total DNA fluorescence signal.

## Supporting Information

Figure S1 Monitoring cellular β-catenin accumulation in response to the Wnt/β-catenin pathway inhibitor ICG-001. L-cells were treated with Wnt3a (50 ng/ml) in the absence or presence of 10 µM ICG-001 for the indicated times. Each point represents mean±SEM (n = 4).
(0.27 MB TIF)

Figure S2 Detection of cellular β-catenin in osteosarcoma U2OS cells, and SW480 and HCT15 colon cancer cell lines. (A) Titration of β-catenin mAb. U2OS cells were incubated in the absence of presence of Wnt3a (50 ng/ml) for 24 h. (B) Detection of β-catenin in SW480 and HCT15 cell lines. m denotes mouse species.
(0.20 MB TIF)
