# Supplementary figures and images for: Kinetics of Wnt-Driven β-Catenin Stabilization Revealed by Quantitative and Temporal Imaging
Source: PLoS One. 2008 Oct 22;3(10):e3498. doi: 10.1371/journal.pone.0003498 (PMC2570213; doi:10.1371/journal.pone.0003498)

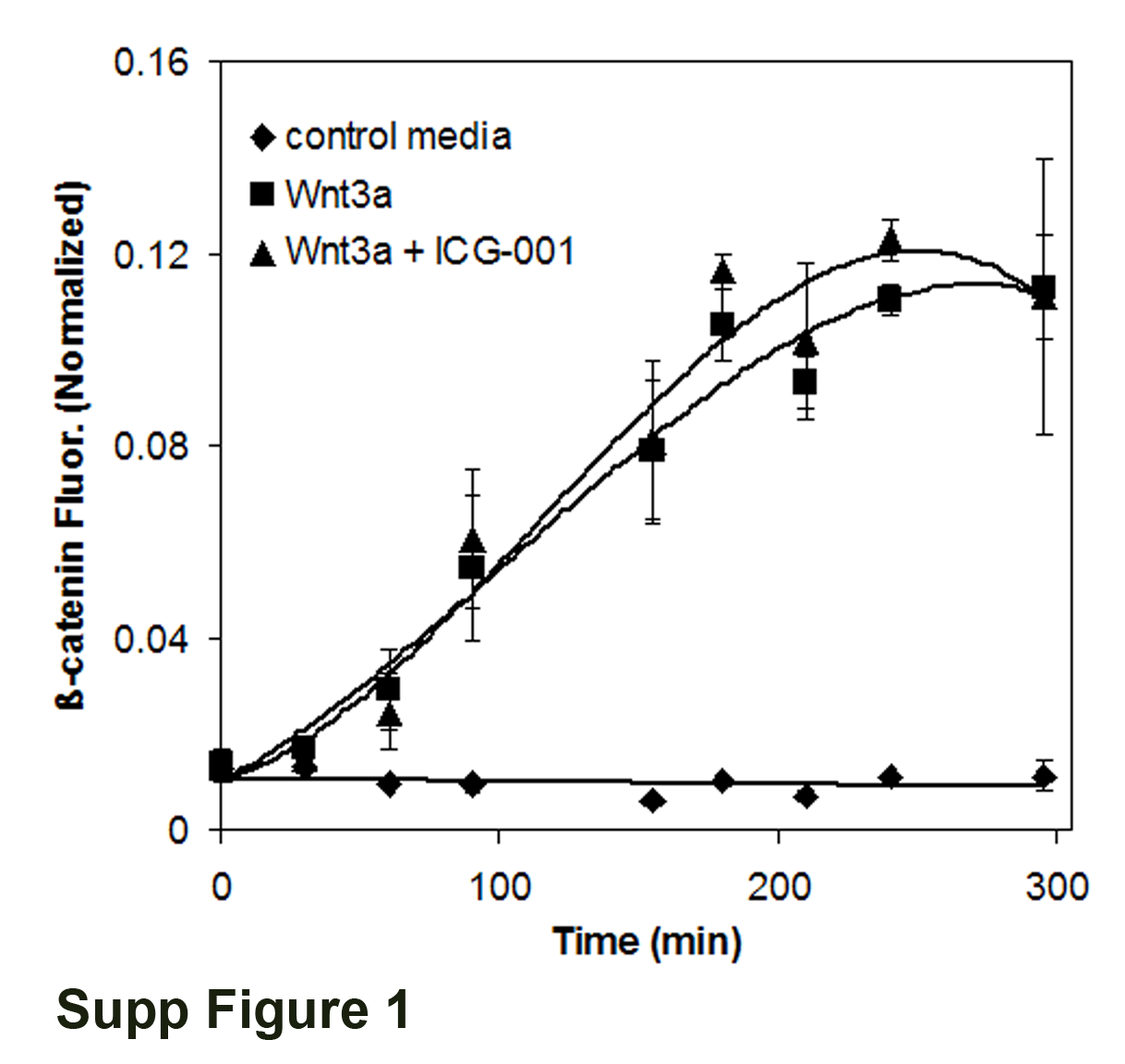

Supplement: Figure S1 — Monitoring cellular β-catenin accumulation in response to the Wnt/β-catenin pathway inhibitor ICG-001. L-cells were treated with Wnt3a (50 ng/ml) in the absence or presence of 10 µM ICG-001 for the indicated times. Each point represents mean±SEM (n = 4). (0.27 MB TIF) [file pone.0003498.s001.tif]

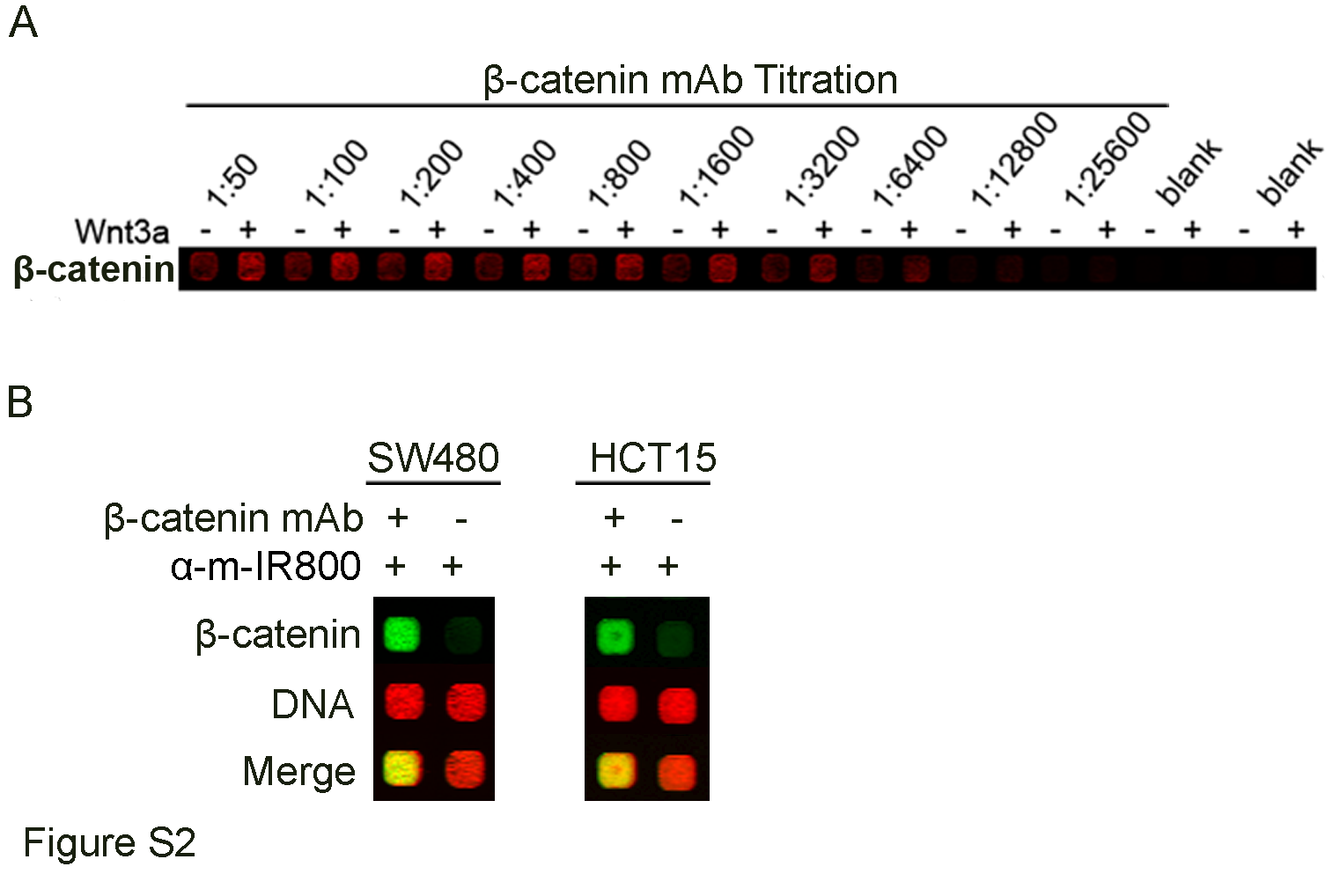

Supplement: Figure S2 — Detection of cellular β-catenin in osteosarcoma U2OS cells, and SW480 and HCT15 colon cancer cell lines. (A) Titration of β-catenin mAb. U2OS cells were incubated in the absence of presence of Wnt3a (50 ng/ml) for 24 h. (B) Detection of β-catenin in SW480 and HCT15 cell lines. m denotes mouse species. (0.20 MB TIF) [file pone.0003498.s002.tif]
